# Distinct Patterns of Cognitive Conflict Dynamics in Promise Keepers and Promise Breakers

**DOI:** 10.3389/fpsyg.2018.00939

**Published:** 2018-06-11

**Authors:** Cinzia Calluso, Anne Saulin, Thomas Baumgartner, Daria Knoch

**Affiliations:** ^1^Department of Social Psychology and Social Neuroscience, Institute of Psychology, University of Bern, Bern, Switzerland; ^2^Department of Business and Management, LUISS Guido Carli University, Rome, Italy

**Keywords:** mouse kinematics, cognitive conflict, social decision-making, promise, inter-individual variability

## Abstract

On a daily basis, we see how different people can be in keeping or breaking a given promise. However, we know very little about the cognitive conflict dynamics that underlie the decision to keep or break a promise and whether this is shaped by inter-individual variability. In order to fill this gap, we applied an ecologically valid promise decision task with real monetary consequences for all involved interaction partners and used mouse tracking to identify the dynamic, on-line cognitive processes that underlie the decision to keep or break a promise. Our findings revealed that on average, the process of breaking a promise is associated with largely curved mouse trajectories, while the process of keeping a promise was not, indicating that breaking a promise is associated with a larger conflict. Interestingly, however, this conflict pattern was strongly shaped by individual differences. Individuals who always kept their promises did not show any signs of conflict (i.e., straight mouse trajectories), indicating that they were not tempted by the monetary benefits associated with breaking the promise. In contrast, individuals who did not always keep their promise exhibited a large conflict (i.e., curved mouse trajectories), irrespective of whether they broke or kept their promise. A possible interpretation of these findings is that these individuals were always tempted by the unchosen decision option – the desire to act in a fair manner when breaking the promise and the monetary benefits when keeping the promise. This study provides the first piece of evidence that there are substantial inter-individual differences in cognitive conflict dynamics that underlie the decision to keep or break promises and that mouse tracking is able to illuminate important insights into individual differences in complex human’s decision processes.

## Introduction

Promises are very common in everyday life and foster social cooperation as well as trust between people (Chen, 2000; Beauchamp, 2003; Friedrich and Southwood, 2011). The role of promises can be traced back to ancient times, when cooperative infrastructures (i.e., laws, courts or police) did not exist, and more basic forms of cooperation agreements where needed to foster trust and partnership in human societies (Fried, 2015). Indeed, economic game studies have found that pre-play communication, i.e., a statement of intent or promise to cooperate, increases the subsequent levels of cooperative behavior (Charness and Dufwenberg, 2006, 2008, 2010; Vanberg, 2008; Battigalli et al., 2013). However, promises cannot only be kept, but also broken. When people face situations in which breaking a promise results in some kind of profit, they may experience a conflict between the temptation to behave selfishly in order to gain a (monetary) benefit on the one hand, and the desire to act in a fair manner on the other. However, there is a large heterogeneity in people’s propensity to follow through on promises that is not well understood. In particular, we know very little about the cognitive conflict dynamics that underlie the decision to keep or break promises.

In order to fill this gap, we need a technique that allows for measuring a potential conflict associated with an individual’s promise keeping and promise breaking decisions. Here, we used mouse tracking because this method continuously tracks the movement of the mouse cursor during the decision process with a fine graded temporal and spatial sensitivity, enabling inferences about cognitive processes during the formation of a decision that are otherwise not observable (Spivey, 2007; Song and Nakayama, 2009; Freeman et al., 2011a).

Although this technique may sometimes be difficult to adopt when studying complex social and psychological research questions (Hehman et al., 2015), an increasing number of studies has applied this method to study the cognitive dynamics of social behavior (Freeman et al., 2008, 2010, 2011b; Duran et al., 2010; Tabatabaeian et al., 2015). These studies show that it is possible to exploit the mouse tracking technique also for these more complex psychological processes because of its ability to reflect the unfolding of an inner cognitive conflict regardless of its exact nature (Freeman et al., 2011a).

As such, this measure of a continuous stream of motor outputs represents a unique tool to determine whether the cognitive process leading to the selection of one of two decision options (e.g., keeping or breaking a promise) is characterized by a conflict and to identify the conflict pattern. A conflict would be indicated by a movement of the mouse cursor that shows attraction (i.e., a curved trajectory) toward the unselected decision option while moving the mouse toward the decision option that is finally selected (Hehman et al., 2015).

**FIGURE 1:**
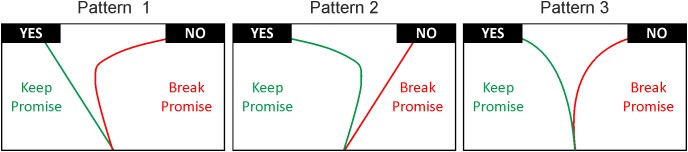
Possible mouse tracking outcomes depending on the source of conflict. According to Pattern 1 conflict mainly arises from the desire to act in adherence to fairness norms, resulting in a strong tendency toward the “YES” response (keeping the promise). According to Pattern 2, the conflict is mainly driven by the temptation to gain (monetary) benefits, resulting in a strong tendency toward the “NO” response (breaking the promise). Finally, according to Pattern 3, trajectories show signs of conflict when participants keep and break the promise, resulting in curved trajectories during both decision processes. The process patterns, however, may be influenced by inter-individual variability.

We hypothesized three possible patterns of results (see **Figure 1** for visualization). Firstly, the conflict associated with keeping vs. breaking a promise may mainly be driven by the desire to act in a fair manner. That means all mouse trajectories associated with breaking a promise should be sharply curved, indicating a high attraction toward the promise keeping option, while all promise keeping responses should yield fairly straight mouse trajectories (i.e., the option to keep the promise is favored by the cognitive process; Pattern 1). Secondly, it may be exactly the other way around. That is, the conflict arises from the temptation to gain a higher monetary outcome implying that the cognitive process favors the option to break the promise (Pattern 2). Thirdly, the cognitive process may not be characterized in terms of favoring either option at all. Instead, it may be characterized by a decision process that is always attracted by the unselected decision option, indicating a conflicting decision process when keeping and breaking the promise (Pattern 3). Importantly, if inter-individual variability shapes the decision process, different participants may in fact show different process patterns. That is, some people may for example show Pattern 1, and others Pattern 2 or 3.

## Materials and Methods

### Participants

Forty-eight right-handed and healthy students (27 females) from the University of Bern took part in the study. They had a mean handedness score of 13.51 (SD ± 2.30; in the scale used in this study a score of 13 can be interpreted as 100% right-handedness and a score of 39 as 100% left-handedness, see Chapman and Chapman, 1987). The final sample comprised 47 participants after the exclusion of one male participant due to technical problems in data recording. This study was approved by the local ethics committee of the Faculty of Human Sciences (University of Bern, Switzerland, Nr. 2015-11-00003). All subjects gave written informed consent in accordance with the Declaration of Helsinki, and were informed of their right to discontinue participation at any time. Participants received 20 Swiss francs (CHF) for participating in addition to the money earned during the Promise Decision task.

### Promise Decision Task

The paradigm used in this study is a Promise Decision task, developed to explore promise keeping and promise breaking decisions in an ecologically valid situation with real consequences for all involved interaction partners (Baumgartner et al., 2009, 2013). One trial in this task involves two anonymously interacting participants, one in the role of an investor and one in the role of a trustee. For the purpose of this study, we focused on the role of the trustee because only participants in this role can keep or break promises. The trustee has to make a promise prior to a series of three subsequent payback trials. The trustee indicates whether he/she plans to “*always”* or “*sometimes”* share the available money with the investor. At the beginning of each payback trial, the investor receives an endowment of 2 monetary units (MUs, 1 MU = 0.5 CHF) and is informed about the trustee’s promise decision. Based on this information the investor has to decide whether he/she wants to trust the trustee by investing his/her endowment, or whether to keep it for him/herself. If the investor decides to keep the endowment, he/she receives 2 MUs while the trustee receives nothing. Conversely, if the investor decides to send the money to the trustee, the experimenter multiplies the amount by five. Thus, the trustee receives 10 MUs. He/she can now decide whether to pay back half of the money to the investor or not (payback trials). If he/she decides to actually pay back half of the money, the investor and the trustee each earn 5 MUs. However, if the trustee decides to keep the money the trustee earns 10 MUs and the investor earns nothing.

**FIGURE 2:**
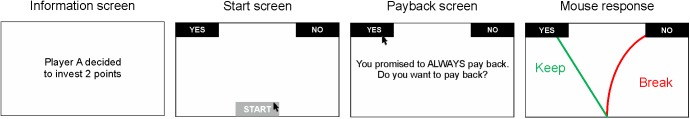
Depicted is one payback trial of the Promise Decision task from the trustee’s perspective. Each payback trial started with an information screen that informed the trustee about the investor’s decision. In case that the investor invested the two MUs, a start screen appeared and the trustee had to press the “START” button. After clicking the “START” button, the trustee was reminded of his/her promise and was asked whether he/she wants to pay back half of the MUs. The trustee then had to decide whether he/she actually wants to pay back (“YES”) or not (“NO”) by moving the mouse toward and finally clicking on the corresponding decision option at the top of the decision screen. Each participant completed a total of 24 such payback trials that were played with different and anonymous investors.

Participants gave their promises prior to three subsequent payback trials eight times, resulting in a total of 24 payback trials for the participants in the role of the trustee (for visualization of one payback trial, see **Figure 2**). These payback trials were played with different, anonymous interaction partners in the role of an investor in order to exclude reputation effects and strategic spillovers across payback trials. Unknown to the participants, the decisions of the investors were preselected based on a pilot study in order to make sure that all participants experienced the same amounts of trusting decisions of the investors. Of the 24 payback trials, 3 trials were decisions in which the investors did not trust the trustee and thus the participants could not make a payback decision in these trials. At the end of the experiment, four trials were randomly selected and the sum earned in these trials was paid in addition to the show-up fee. The experiment was run in z-Tree (Fischbacher, 2007) and interfaced with the software package OpenSesame (Mathôt et al., 2012) in order to allow for the recording of mouse movements (sampling rate = 100 Hz). To ensure a proper recording of mouse movements, participants were instructed to press a *start* button, positioned at the bottom center of the screen. Then participants were free to choose a decision option by clicking on the corresponding response button. Participants were also explicitly instructed to begin their movements early and were warned after slow trials (movement started later than 1500 ms after trial onset). The response buttons were equidistantly positioned from the *start* button at the top left and top right of the screen. The mapping between response options and response positions was counterbalanced across participants. Please see **Figure 2** for an example of a payback trial.

### Data Analysis

#### Kinematics and Temporal Indices

Mouse trajectories (x- and y- coordinates) associated with participants’ choices were recorded at a sampling rate of 100 Hz during the payback decision. Due to differences in response times, each trial resulted in a different number of x-y-coordinate pairs. To account for these differences, trajectories were time-normalized prior to statistical analyses using a publicly available python code (Travers, 2013) which is based on a standard routine (Spivey, 2007; Freeman et al., 2011a). In that vein, trajectories were first rescaled into a standard coordinate space. In this new space, the top-left corner of the screen corresponds to “-1; 1.5”, the top-right corner to “1; 1.5”, and the position of the *start* button to “0, 0”. Then the duration of the trajectory movements was normalized by re-sampling the time vector into 101 time-bins using linear interpolation to allow averaging and comparisons across multiple trials. Additionally, the endpoints of all trajectories were flipped in the same direction (i.e., toward the right-side response button). These normalized trajectories were used to compute two parameters based on the mouse trajectories: the maximum deviation (MD) and the area under the curve (AUC). The MD is the largest perpendicular deviation between the real trajectory and the theoretical trajectory (i.e., straight line connecting each trajectory’s start and endpoints) out of all time steps. The AUC is the geometric area between the real and theoretical trajectory. Although these two measures are different from a mathematical and geometrical standpoint, they both provide an index of spatial attraction toward the concurrent not-selected option (i.e., high values of MD and AUC indicate a high spatial attraction by the unselected response option while moving the mouse toward the selected option) (Spivey, 2007; Freeman and Ambady, 2010; Freeman et al., 2011a). Further, prior studies have shown no change in the results if MD and AUC are interchangeably used (Freeman et al., 2008; Calluso et al., 2015).

In addition, a series of temporal measures were computed. Firstly, the *response time* (in ms) denotes the time from the moment the *start* button was clicked until the response was given. Secondly, the *initiation time* (in ms) is the amount of time from the moment the participant clicked on the *start* button until they actually initiated the movement of the mouse cursor. Finally, the *motion time* (in ms) is calculated as the amount of time after the mouse movement was initiated until the participant clicked on the final answer. It is important to note that even if the traditional response time measure includes both the initiation and motion times, these three measures reflect different times at which uncertainty/conflict may become apparent. For example, if conflict arises during early stages of processing, it would be reflected by longer latencies in initiation time, while later on in the decision process it would be reflected in the motion time (e.g., because of a high number of changes in direction).

#### Statistical Analysis

We focused in our analyses on payback trials that were preceded by an always promise because deciding to not pay back after an always promise can unambiguously be identified as breaking a promise and deciding to pay back after an always promise can unambiguously be identified as keeping a promise. Conversely, when a participant promised to sometimes pay back it is not clear whether keeping this promise corresponds to paying back once or paying back twice in the three trials following that promise and thus it is *not* possible to unambiguously identify keep and break trials. Thus, we focused on the clearly interpretable payback decisions that followed always promises. In this regard, please note that the majority of payback trials (75%) were preceded by an always promise. Payback trials were first classified as *keep* or *break* trials. Keep trials were those trials in which participants first promised to always pay back and then decided to return half of the MUs to the investors (i.e., *always* – *yes* responses). In contrast, break trials were those trials in which participants promised to always return half of the MUs but then they decided not to follow through on that promise (i.e., *always – no* responses). Further, for every participant, the return rate (total percentage of keep trials following an always promise) was calculated as an index of the overall payback behavior.

In a next step, a series of linear mixed-effects models analyses with fully specified random effects structure were conducted, independently using mouse kinematics (MD and AUC) as well as the temporal measures (response time, moving time, initiation time) as dependent variables, while the response type (break vs. keep trials) was used as fixed effect. This approach allows us to account for potential inter-individual differences in baseline motor behavior. Further, because 23 of the 47 participants showed no variance in their responses (they decided to keep their promise in 100% of the trials) we conducted an additional series of linear mixed-effect models (LMMs) analyses with the same structure described above, discarding this subgroup of participants.

In order to elucidate the role of inter-individual variability in overall behavior regarding the potential conflict involved in promise keeping vs. promise breaking the sample was divided into two groups based on the individual return rate. The first group comprised those participants whose return rate was equal to 100%, i.e., participants who kept their promise of always paying back in 100% of the payback trials (promise keepers; *N* = 23). The second group comprised all those participants who did not always keep their promise of always paying back, i.e., their return rate was lower than 100% (promise breakers; *N* = 24). To test whether these two groups differ with respect to their promise behavior, a repeated measures ANOVA was conducted on the percentage of always and sometimes promises, using the promise (*always* vs. *sometimes*) as within-subject factor, and the group (promise keepers vs. promise breakers) as between-subject factor.

In a series of LMM analyses, the kinematics and temporal measures associated with keep trials of promise keepers were compared to those associated with keep and break trials of promise breakers. Notably, three of the twenty-four promise breakers never kept their promises across the entire experiment (return rate *m* = 0%). Thus, these three participants could not be included in the analysis that comprised keep trials. These analyses were conducted using the kinematics and temporal measures as dependent variables, while the group (i.e., promise breakers vs. promise keepers) was included as fixed effect. Analyses were run separately for each dependent variable.

Additionally, to pin down the pattern of conflict in promise breakers, we compared keep and break trials in this group of participants (*N* = 21). Hence, a series of LMMs analyses was conducted on the kinematics and temporal measures, using the response type (break vs. keep trials) as fixed effect. Again, three of the twenty-four participants belonging to the group of promise breakers had to be excluded from this analysis because they never kept their promises (return rate *m* = 0%). Since the group of promise breakers yielded a large inter-individual variability with respect to the individuals’ return rates, we conducted additional follow-up LLMs analyses which were restricted to those promise breakers who displayed a comparable amount of promise keep and break decisions (return rate: *m* = 56% ± 11%, *N* = 14).

All the LMMs analyses described in this section were conducted using the intercepts for subjects, trials, as well as the random slopes of the fixed effects as random effects.

Finally, since promise breakers are characterized by large inter-individual variability, an additional set of analyses was conducted to elucidate whether the frequency of promise keeping decisions (i.e., return rate) had an impact on the curvature of mouse trajectories associated with keep and break trials. In that vein, we first computed an index of cognitive conflict for each of the kinematics measures (i.e., MD and AUC). This index was calculated as the difference between the MD/AUC associated with the break trials minus the one associated with keep trials [i.e., MD _index_ = MD *_break_* – MD *_keep_*; AUC _index_ = AUC *_break_* – AUC *_keep_*]. Then, robust linear regressions (Holland and Welsch, 1977; O’Leary, 1990) were conducted independently using the MD and AUC indices as dependent variables, and the return rate as a regressor. Again, three of the twenty-four participants belonging to the group of promise breakers had to be excluded from these analyses because they never kept their promises (return rate *m* = 0%), thus, these analyses were conducted on 21 promise breakers.

All the LMM analyses described above were also conducted controlling for possible gender effects. Thus, we re-ran all LLM analyses and added gender as a covariate. Further, an additional series of analyses was conducted to test whether gender may moderate the effect of response type (break vs. keep trials) or group (promise breakers vs. promise keepers) on the kinematics and temporal measures. To this aim, a series of LMM analyses was conducted including a gender by group or gender by trial type (keep vs. break) interaction term (in these analyses gender was also included as a single predictor). Notably, all findings reported in the paper hold if we control for gender in our analyses. Furthermore, there is also no evidence for an interaction effect. All *p*-values of the interaction terms of gender by trial type or gender by group are not significant (all *p* > 0.13).

## Results

### Descriptive Statistics

**FIGURE 3:**
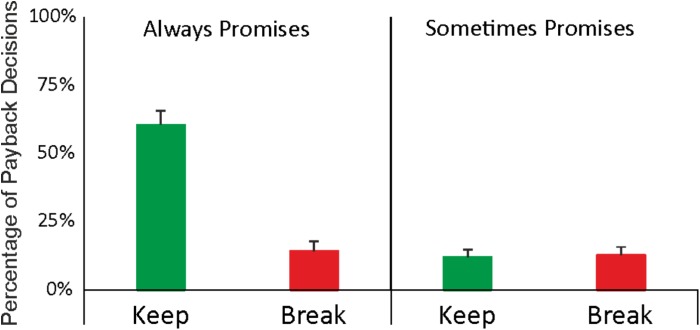
Average percentages of keep (green bars) and break (red bars) decisions as a function of promise decisions (“ALWAYS” vs. “SOMETIMES”). Overall, 75% of the promise decisions were “ALWAYS” decisions whereas 25% were “SOMETIMES” decisions.

The participants choose the always promise (*m* = 75%) with higher frequency than the sometimes promise (*m* = 25%; *t* = 5.75, *p* < 0.001). Furthermore, participants exhibited a higher number of *yes (keep)* compared to *no (break)* decisions when they had promised to always pay back [*always* – *yes* (*m* = 61 %) vs. *always* – *no* (*m* = 14%); *p* < 0.001]. Conversely, this difference was not observed when participants had promised to sometimes pay back [*sometimes* – *yes* (*m* = 12%) vs. *sometimes* – *no* (*m* = 13%); *p* = 0.9, see **Figure 3**].

The following mouse tracking analyses focus on those payback trials that were preceded by an always promise because only these trials can be unambiguously classified as promise keeping or promise breaking trials.

### Testing for the Existence of a Conflict

**FIGURE 4:**
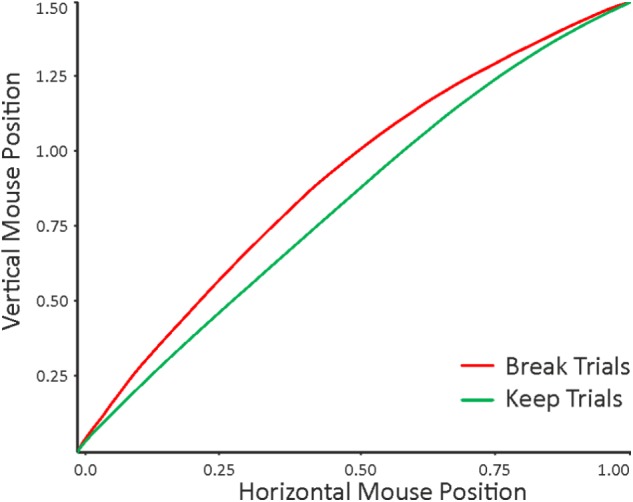
Mean mouse trajectories associated with keep trials (i.e., promise: always; payback: yes; green line) and break trials (i.e., promise: always; payback: no; red line). The trajectories were normalized into a standard space and flipped into the same direction prior to statistical analyses. The point (0, 0) corresponds to the position of the “START” button and the point (1, 1.5) to the decision button (i.e., “YES” or “NO”). One can see that on average, break trials are associated with a larger Maximum Deviation than keep trials.

In a first step, we investigated whether there exists a conflict at all when participants decide whether to keep or break a promise. Hence, we tested whether the mouse trajectory during the decision to pay back or not was significantly associated with the decision outcome (i.e., response type: keeping the promise vs. breaking the promise). In that vein, we used a series of LMMs analyses with the mouse kinematics (i.e., MD, AUC) and temporal measures (i.e., response time, initiation time, and motion time) as dependent variables and response type as independent variable (for details, see methods section). The analyses revealed that response type was significantly associated with both the MD (β = -0.05, *p* < 0.001) and the AUC (β = -0.05, *p* < 0.001), indicating that break trials were associated with a stronger tendency toward the unselected response compared to keep trials (**Figure 4**). Such findings are in line with the hypothesized Pattern 1 (see **Figure 1**), indicating a stronger conflict when breaking a promise compared to when keeping a promise. That said, these results support the existence of a conflict during the decision whether to keep or break a promise and the cognitive process underlying this conflict appears to favor the option to keep the promise.

It is interesting to note that none of the temporal measures (response time: *p* = 0.77, initiation time: *p* = 0.69, motion time: *p* = 0.58) were able to significantly model the response type. Thus, if we only had acquired response time data, we would not have been able to detect the conflict in break trials. An overview of the results for the linear mixed-effects analyses conducted on the kinematic and temporal measures is provided in **Table 1**.

**Table 1:** Overview of the results of the mixed-effect models analyses testing the effect of response type (keep vs. break trials) on the kinematic and temporal measures.

	Response type			
	Coeff.	*SE*	*t* value	*p*-value
Maximum Deviation	-0.05	0.02	-3.35	<0.001
Area Under the Curve	-0.05	0.02	-3.41	<0.001
Response time	-20.18	67.75	-0.30	0.77
Initiation time	7.17	18.17	0.39	0.69
Motion time	-3E+01	6E+01	-6E-01	0.58

*Coefficient estimates from mixed-effects models.*

Interestingly, the same results were also replicated in the LLM analyses conducted excluding the 23 trustees characterized by no variance in their decision (i.e., return rate = 100%). Indeed, as in the previous analyses, findings revealed a significant effect of response type on MD (β = -0.05, *p* < 0.01) and AUC (β = -0.05, *p* < 0.01), while no significant results were found for the temporal measures (response time: *p* = 0.94; initiation time: *p* = 0.50; motion time: *p* = 0.75). An overview of the results for the linear mixed-effect models analyses conducted on the kinematic and temporal measures is provided in **Table 2**.

**Table 2:** Overview of the results of the mixed-effect models analyses testing the effect of response type (keep vs. break trials) on the kinematic and temporal measures, excluding the 23 trustees that showed no variance in their decision.

	Response type			
	Coeff.	*SE*	*t* value	*p*-value
Maximum Deviation	-0.05	0.01	-2.99	<0.01
Area Under the Curve	-0.05	0.01	-3.00	<0.01
Response time	-5.56	75.01	-0.07	0.94
Initiation time	15.33	22.77	0.67	0.50
Motion time	-20.99	66.06	-0.31	0.75

*Coefficient estimates from mixed-effects models.*

### Conflict During the Decision Shaped by Inter-individual Differences

**FIGURE 5:**
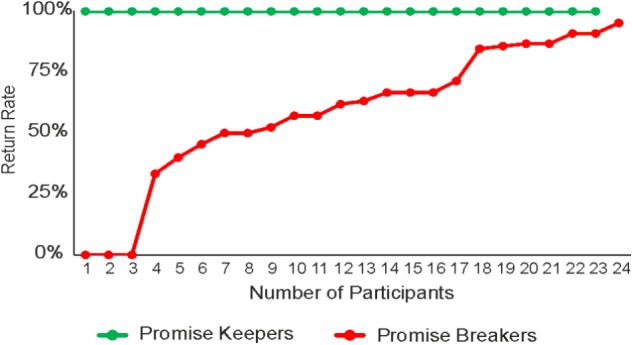
Mean return rate of promise keepers (green line) and promise breakers (red line).

The results obtained in the previous analyses suggest that (i) a conflict exists when people are faced with the decision to keep or break a given promise, and (ii) the cognitive process associated with this conflict favors the option to keep the promise. However, it remains an open question whether everyone has the same conflict pattern or whether this conflict pattern is shaped by inter-individual differences. In order to answer this question, participants were divided, based on their return rate, in two groups: promise keepers (return rate = 100%; **Figure 5** green line) and promise breakers (return rate = 58% ± 28%; **Figure 5** red line). In a first analysis, a repeated measures ANOVA was conducted to check whether promise keepers and promise breakers differ in their chosen promise level. The results showed a significant main effect of promise (always > sometimes; *F*_1,45_ = 33.00, *p* < 0.001). Importantly however, no promise by group interaction was observed (*F*_1,45_ = 1.52, *p* = 0.22). This finding indicates that promise keepers and promise breakers did not differ in the chosen promise level (both groups chose “always” most of the time). Thus, differences in keep and break decisions and conflict patterns cannot be attributed to differences in the chosen promise level.

Thus, in the following, mouse trajectories and temporal measures of promise keepers and promise breakers were compared by conducting a series of LMM analyses. Please note that a comparison of break trials in promise keepers and break trials in promise breakers is not possible since promise keepers do not have break trials.

**FIGURE 6:**
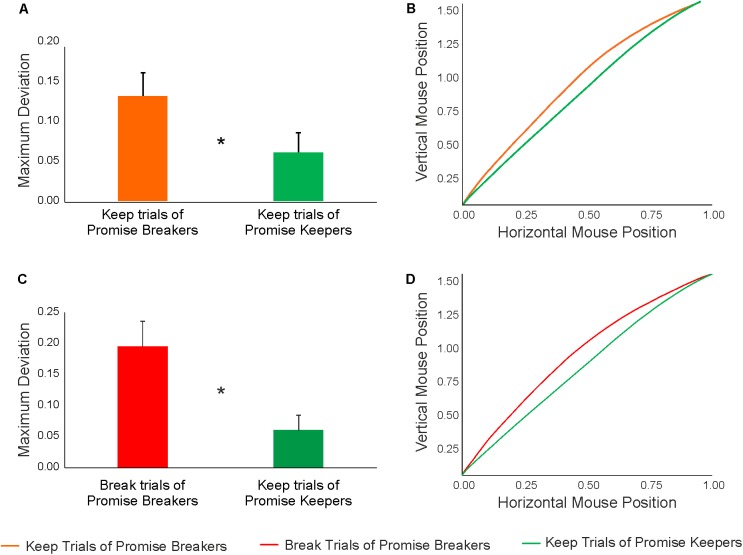
**(A)** Results of the LMM analysis conducted to compare the MD associated with keep trials of promise keepers (*N* = 23) vs. keep trials of promise breakers (*N* = 21). **(B)** Mean mouse trajectories of keep trials of promise keepers (green line) and keep trials of promise breakers (orange line). **(C)** Results of the LMM analysis conducted to compare the MD associated with keep trials of promise keepers (*N* = 23) vs. break trials of promise breakers (*N* = 24). **(D)** Mean mouse trajectories of keep trials of promise keepers (green line) and break trials of promise breakers (red line). Statistically significant (*p* < 0.05) comparisons are marked by an asterisk.

In a first step, keep trials of promise keepers and keep trials of promise breakers were compared. Note that three of the twenty-four participants in the group of promise breakers could not be included in these analyses because they never kept their promise (i.e., they do not have any keep trials). The results revealed that the two groups significantly differed in both kinematic measures (MD: β = -0.05, *p* < 0.05; AUC: β = -0.05, *p* < 0.05, see **Figure 6A**), indicating that promise breakers yielded a stronger conflict than promise keepers, even when keeping their promise. Along the same lines, within the temporal measures, a significant group effect was observed in the response time (β = -325.20, *p* < 0.05), in the initiation time (β = -74.59, *p* < 0.01) and in the motion time (β = -243.99, *p* < 0.05). These results suggest that promise breakers were characterized by higher curvature, higher hesitation, longer motion and response times, even when they kept the promise given beforehand (**Figure 6B**). The detailed results of these analyses are reported in **Table 3**.

**Table 3:** Overview of the results of the mixed-effect models analyses testing the effect of group (promise keepers vs. promise breakers) on the kinematics and temporal measures associated with keep trials.

	Group: Promise Keepers vs. Promise Breakers			
	Coeff.	*SE*	*t* value	*p*-value
Maximum Deviation	-0.05	0.02	-2.08	<0.05
Area Under the Curve	-0.05	0.03	-2.07	<0.05
Response time	-325.20	133.00	-2.45	<0.05
Initiation time	-74.59	22.41	-3.33	<0.01
Motion time	-243.99	113.97	-2.14	<0.05

*Coefficient estimates from mixed-effects models.*

In a second step, keep trials of promise keepers and break trials of promise breakers were compared (in these analyses all twenty-four participants in the group of promise breakers were included). The results revealed that the two groups significantly differed in both kinematic measures (MD: β = -0.12, *p* < 0.01; AUC: β = -0.13, p < 0.01) (**Figure 6C**). That is, promise breakers yielded a stronger conflict when breaking their promise compared to promise keepers when keeping their promise. Within the temporal measures, a nearly significant group effect was observed in the response time (β = -309.90, *p* = 0.09), while no group effect was observed in the initiation time (β = -309.90, *p* = 0.30) and in the motion time (β = -230.80, *p* = 0.13). This means that promise breakers displayed more curved trajectories (i.e., stronger conflict) and longer response times when breaking a promise. Conversely, participants who always kept their promise displayed straight trajectories (corresponding to Pattern 1) and faster response times (**Figure 6D**). The detailed results of these analyses are reported in **Table 4**.

**Table 4:** Overview of the results of the mixed-effect models analyses testing the effect of the group (promise keepers vs. promise breakers) on the kinematics and temporal measures associated with keep trials of promise keepers and break trials of promise breakers.

	Group: Promise Keepers vs. Promise Breakers			
	Coeff.	*SE*	*t* value	*p*-value
Maximum Deviation	-0.12	0.05	-2.59	<0.01
Area Under the Curve	-0.13	0.04	-2.55	<0.01
Response time	-309.90	181.20	-1.71	0.09
Initiation time	-75.91	72.76	-1.04	0.30
Motion time	-230.80	154.70	-1.49	0.13

*Coefficient estimates from mixed-effects models.*

### Conflict in Promise Breakers

The previous analyses showed that participants in the group of promise keepers did not show significant signs of conflict or hesitation during their decision to keep the promise, whereas promise breakers showed significantly higher level of conflict both when keeping and breaking a promise. However, it remains open whether these participants experience an equally large conflict when they break a promise compared to when they keep it. In a final step, to pin down the source of conflict in this group of participants (**Figure 5**, red line), the effect of response type (i.e., keep and break trials) on the kinematic and temporal measures was tested in promise breakers. As previously reported in the methods section, three of the twenty-four participants in this group are not included in this analysis because they never kept their promise (i.e., they do not have any keep trials).

**FIGURE 7:**
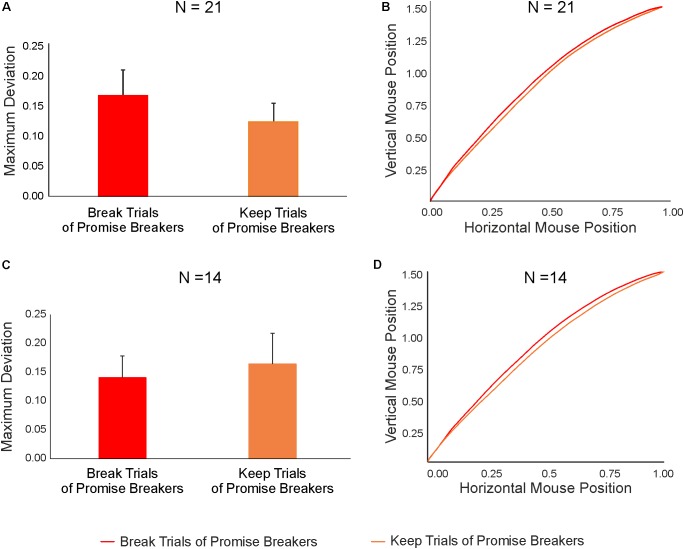
**(A)** Results of the LMM analysis conducted to compare the MD associated with keep and break trials of promise breakers (*N* = 21). **(B)** Mean mouse trajectories of keep (orange line) and break (red line) trials of promise breakers. **(C)** Results of the LMM analysis performed to compare the MD of keep and break trials in a subset of promise breakers with a similar number of promise keep and promise break trials (*N* = 14). **(D)** Mean mouse trajectories of keep trials (orange line) and break trials (red line) of this subset of promise breakers.

Interestingly, these analyses revealed no statistically significant differences between keep and break trials in any of the kinematic (MD: *p* = 0.50; AUC: *p* = 0.53) or temporal measures (response time: *p* = 91; initiation time *p* = 0.83; motion time: *p* = 0.93; **Figures 7A,B**; see **Table 5** for detailed statistical results). This means that in promise breakers, the cognitive process associated with the decision whether to keep or break a promise is likely to adhere to Pattern 3, indicating a similarly strong conflict during both break and keep trials (compare **Figure 1**).

**Table 5:** Overview of the results of the mixed-effect models analyses testing the effect of response type (keep vs. break) on the kinematics and temporal measures in promise breakers.

	Response type			
	Coeff.	*SE*	*t* value	*p*-value
Maximum Deviation	-0.05	0.07	-0.67	0.50
Area Under the Curve	-0.05	0.08	-0.62	0.53
Response time	-23.23	195.04	-0.12	0.91
Initiation time	9.40	44.58	0.21	0.83
Motion time	-34.33	388.50	-0.09	0.93

*Coefficient estimates from mixed-effects models.*

Further, since the group of promise breakers is characterized by a high degree of variability with respect to their return rate (see **Figure 5**, red line), we focused on only those promise breakers with a comparable number of both trial types (i.e., keep and break trials; compare **Figure 5**: participants 4–17 in the group of promise breakers, red line, *N* = 14) in order to ensure that the results obtained in the previous analyses were not affected by the different number of promise breakers’ keep and break trials. Notably, the results of this comparison replicated those reported above. That is, we found no statistically significant differences between keep and break trials in any of the kinematics (MD: *p* = 0.39; AUC: *p* = 0.40) or temporal measures (response time: *p* = 0.88; initiation time *p* = 0.89; motion time: *p* = 0.83; **Figures 7C,D**; see **Table 6** for detailed results).

**Table 6:** Overview of the results of the mixed-effect models analyses testing the effect of response type (keep vs. break) on the kinematics and temporal measures in promise breakers exhibiting a similar number of keep and break decisions (*N* = 14).

	Response type			
	Coeff.	*SE*	*t* value	*p*-value
Maximum Deviation	-0.06	0.07	-0.86	0.39
Area Under the Curve	-0.06	0.07	-0.85	0.40
Response time	-72.44	479.61	-0.15	0.88
Initiation time	14.23	105.91	0.13	0.89
Motion time	-87.04	403.40	-0.22	0.83

*Coefficient estimates from mixed-effects models.*

Finally, an additional set of analyses was conducted to test whether the degree of cognitive conflict in promise breakers was influenced by the frequency of promise keeping (return rate). To this aim, two indices of cognitive conflict (break trials minus keep trials, see Materials and Methods) were independently calculated from the kinematics measures (i.e., MD and AUC) and used as dependent variables in a robust regression with the return rate as predictor. The results of these analyses showed no association between the return rate and the kinematics indices in both MD (β = 0.18, *p* = 0.42) and AUC (β = 0.17, *p* = 0.41). Thus, these findings suggest that the frequency of promise keeping in promise breakers does not have an impact on the conflict experienced during keep and break trials, corroborating the previous analyses.

### Promise Stage

Although not the main focus of the study, we also analyzed whether promise breakers and promise keepers already differed in cognitive conflict dynamics during the promise stage, i.e., when they promised to always pay back the money. Due to the same reasons as outlined above, we focused on the always promises. We conducted linear regression analyses on the spatial and temporal measures of mouse kinematics collected during the promise decisions and used the group (i.e., promise keepers vs. promise breakers) as a categorical predictor (fixed effect). The results showed that the regression analyses did not reach statistical significance in any of the dependent measures (MD: β = -0.03, *t* = -0.75, *p* = 0.46; AUC: β = -0.04, *t* = -1.00, *p* = 0.33; response time: β = -218.95, *t* = -1.05, *p* = 0.30; initiation time: β = -37.38, -0.89, *p* = 0.38; motion time: β = -174.72, *t* = -0.97, *p* = 0.34). These findings suggest that promise keepers and promise breakers do not differ in conflict dynamics during the promise decision. However, please note that we are reluctant to further interpret these results because the design was not optimized for the analyses of the promise decisions. In particular, there are on average only six “always” promise decisions per subject, which yields issues of statistical power and reliability. Thus, these results should be considered exploratory and should only be used to guide future researchers.

## Discussion

Using mouse tracking we were able to continuously index participants’ tentative commitments to two choice alternatives during the decision of whether to keep or break a given promise. We provide new evidence regarding different conflict patterns of promise breakers and promise keepers. Interestingly, promise breakers exhibited a strong conflict during their decisions, regardless of whether they were keeping or breaking a promise (corresponding to Pattern 3 of **Figure 1**). This suggests that they were always tempted by the non-selected decision option regardless of whether they actually kept or broke their promise. In contrast, promise keepers did not show any signs of conflict during keeping the promise (corresponding to Pattern 1 of **Figure 1**). This suggests that promise keepers were not tempted by the monetary gain associated with breaking the promise. Additionally, the fairly straight trajectories exhibited by promise keepers suggest a strong tendency toward keeping the promise. Since in the present paradigm, participants were free to keep or break their promises, it is not possible to obtain promise breaking decisions from those participants who showed a strong preference for keeping the promise. However, in line with the predictions of Pattern 1, one would expect sharply edged trajectories for decisions in which these participants (would have to) break their promise.

Individuals who did not always keep their promise (i.e., promise breakers) exhibited a conflict, irrespective of whether they broke or kept their promise. This conflict pattern allows two different interpretations. On the one hand, one could argue that the nature of conflict is different when promise breakers keep and break a promise. When breaking a promise, they might experience a moral conflict because they are aware of the fact that they behave in an unfair manner. When keeping a promise, they might experience an economic conflict because they forego monetary benefits, i.e., they make a decision that is against their economic self-interest. On the other hand, an alternative interpretation could also be that the observed conflict pattern reflects people’s indecisiveness. These two interpretations are not necessarily incompatible, but can be reconciled since such indecisiveness might be based on the same underlying decision conflicts: a moral decision conflict when breaking the promise and an economic decision conflict when keeping the promise. However, the present study cannot exclude the possibility that the cognitive conflict associated with the mouse trajectories of promise breakers arises from aspects that are unrelated to promise keeping or promise breaking behavior *per se*. That is, the curved mouse trajectories exhibited by promise breakers in keep and break trials cannot be undoubtedly interpreted as originating from the desire to act in a fair manner or for monetary benefit, respectively. Future investigations may be able to clarify this point.

Further, if we had used a more traditional measure of conflict, i.e., response times, our conclusions would be limited. First, if we only had measured response time, we would not have been able to detect the conflict in break trials. Second, there are findings in our study where both the mouse trajectories and the response time measure showed a similar negative finding, that is, none of these measures differentiated between keep trials and break trials in promise breakers. At first sight, this indicates no advantage of mouse tracking. However, at second sight, the advantage of mouse tracking becomes clear. Decisively, in contrast to response time, mouse tracking is able to reveal the psychological process while it unfolds during deliberation (Spivey, 2007; Freeman et al., 2011a). In the present study, the individual differences were manifest exactly in this unfolding process. Only by analyzing the shape of the mouse trajectories, we were able to learn that promise breakers have a conflict both in keep and break trials, and thus ruling out the alternative interpretation that there is no conflict at all in this group. If we only had measured response time, we would not have been able to differentiate these two interpretations. Together, these findings suggest that although response time measures are often an important and illuminating component of a decision, mouse tracking measures seem to be able to capture additional variance that is distinct from the variance revealed by response time measures.

There are only very few previous studies examining the phenomenon of promise breaking and keeping in a social situation that involves real consequences and where participants were free to keep or break the promise (Baumgartner et al., 2009, 2013). Moreover, only one of these studies explored the decision process of this phenomenon. This study used fMRI to examine the neural correlate of promise breaking and keeping (Baumgartner et al., 2009). In line with the current results, the findings of this study suggested the existence of a conflict during the act of breaking a promise by showing stronger activation of brain regions that had been implicated in conflict detection and resolution (anterior cingulate cortex, ACC and dorsolateral prefrontal cortex, DLPFC; Carter, 1998; Botvinick et al., 1999, 2001; Miller and Cohen, 2001). Similar results have been obtained in a related research field, i.e., deception/dishonest behavior. A consistent finding of studies in this field (Priori et al., 2008; Greene and Paxton, 2010; Kireev et al., 2017; Sun et al., 2017, for a review see Abe, 2011) is that the deceptive act goes along with increased activation of brain regions involved in conflict detection and resolution, as the ACC and DLPFC. Interestingly, some brain studies have also provided evidence for the opposite pattern (e.g., Zhu et al., 2014; Maréchal et al., 2017). For example, a study showed that patients with damages to the DLPFC show less honest behavior compared to patients with lesions in other areas and healthy subjects (Zhu et al., 2014) and another study (Maréchal et al., 2017) indicated that enhanced excitability of the DLPFC by means of brain stimulation increases honest behavior compared to sham stimulation and stimulation that reduced the excitability of the DLPFC. Moreover, studies employing a behavioral approach have shown similar discrepancies. While most studies demonstrated that dishonest behavior was associated with increased reaction times (e.g., Spence et al., 2001; Nuñez et al., 2005), there also exists a number of behavioral studies that have reported the opposite pattern of results (e.g., Shalvi et al., 2012; Tabatabaeian et al., 2015; for a review see Bereby-Meyer and Shalvi, 2015). Although speculative and only in a related research field, the findings of the current study might help to understand the discrepancies in the mentioned studies on dishonest behavior. By taking into account different behavioral types (promise breakers vs. promise keepers), we found evidence for different conflict dynamics in different behavioral types. These divergent conflict dynamics suggest that future studies on dishonest behavior could benefit from an integrative perspective that takes different behavioral types into account.

In sum, using mouse tracking, the current study is able to elucidate the cognitive processes underlying the conflict, which arises during the decision to keep or break a promise given beforehand. Firstly, promise keepers displayed a significantly smaller temptation by the dishonest option than did promise breakers. Secondly, promise breakers were generally more tempted by the non-chosen decision option and not specifically by keeping or breaking their promise, as implicated by equally curved trajectories for promise keep and promise break decisions. Hence, the present study provides the first objective evidence that the conflict dynamics underlying the decision to keep or break a promise is strongly shaped by inter-individual differences.

## Author Contributions

CC, AS, TB, and DK designed the research. CC, AS, and TB collected the data. CC, AS, TB, and DK analyzed the data. CC, AS, TB, and DK wrote the paper.

## Conflict of Interest Statement

The authors declare that the research was conducted in the absence of any commercial or financial relationships that could be construed as a potential conflict of interest.
